# Supplementary material for: A systematic benchmark of machine learning methods for protein–RNA interaction prediction
Source: Brief Bioinform. 2023 Aug 26;24(5):bbad307. doi: 10.1093/bib/bbad307 (PMC10516373; doi:10.1093/bib/bbad307)
Supplement: BIB-23-0185-Horlacher-et-al_SUPP-FIGURES_bbad307 [file bib-23-0185-horlacher-et-al_supp-figures_bbad307.docx]

Supplementary Figures


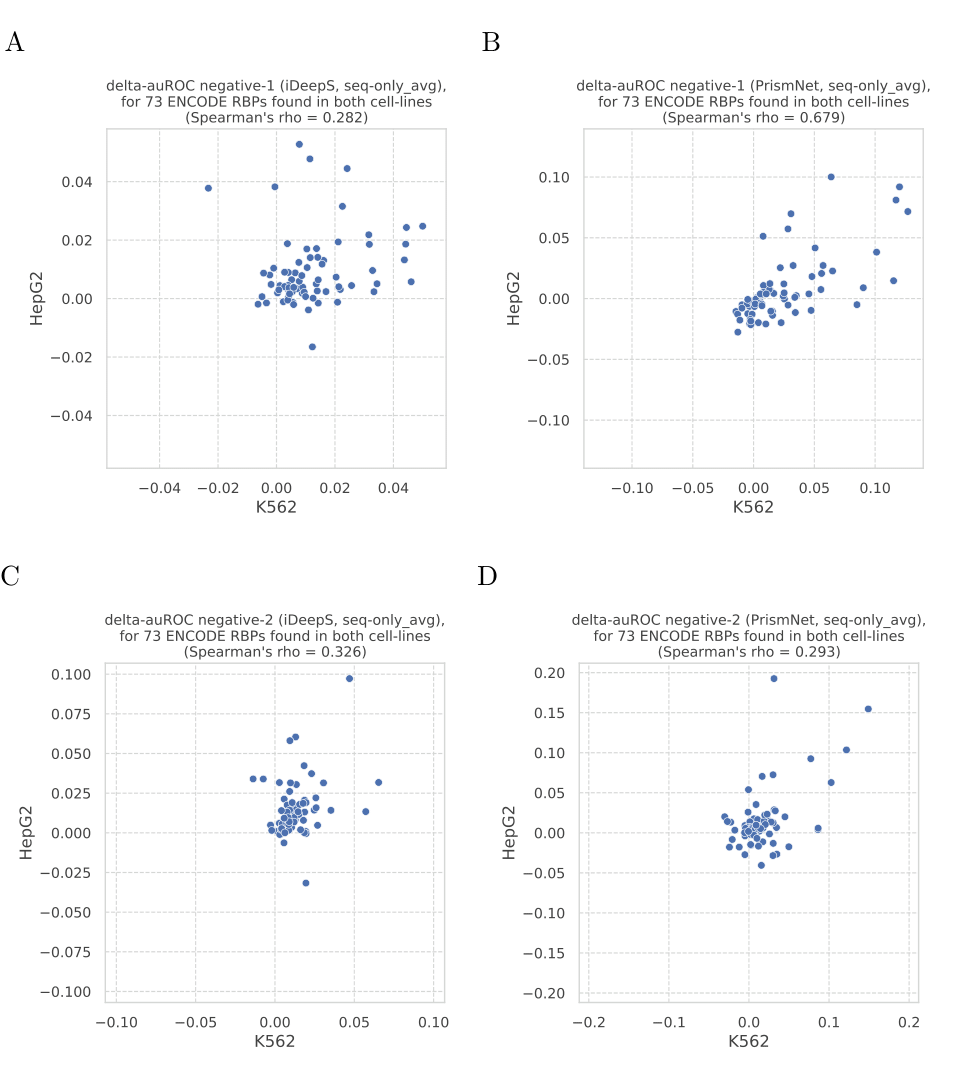


Supplementary Figure 1: Correlation of delta-auROC (increase in performance) from sequence to sequence + structure models between cell types of ENCODE eCLIP experiment. The increase in performance is consistent across negative sets and tools, suggesting that similar structure-sensitive RBPs are picked up across cell types.


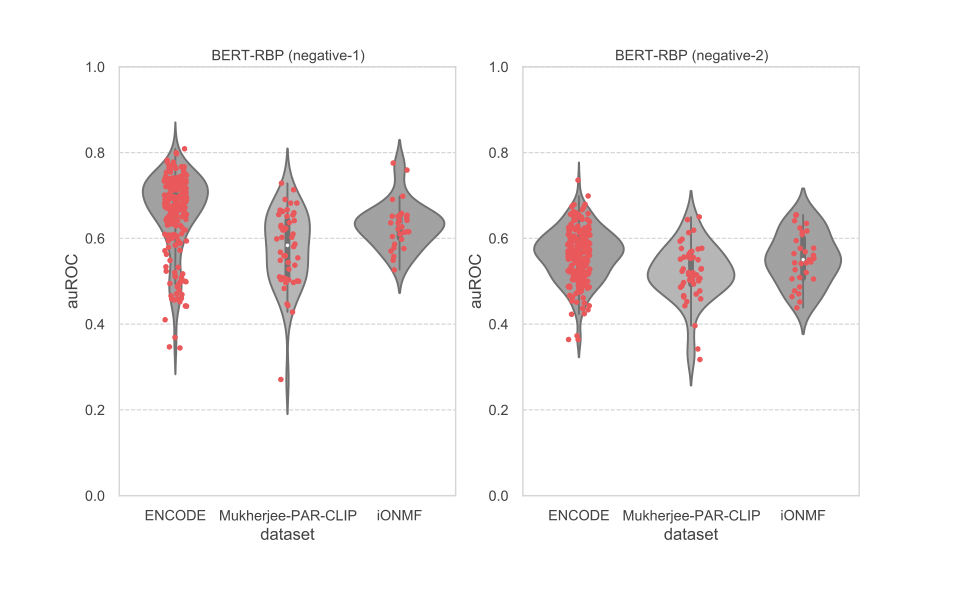


Supplementary Figure 2: Initial results on BERT-RBP, trained with default parameters. Several models showed poor or no convergence, with a performance close to the random baseline. After consulting the authors, BERT-RBP was re-trained with modified hyperparameters.


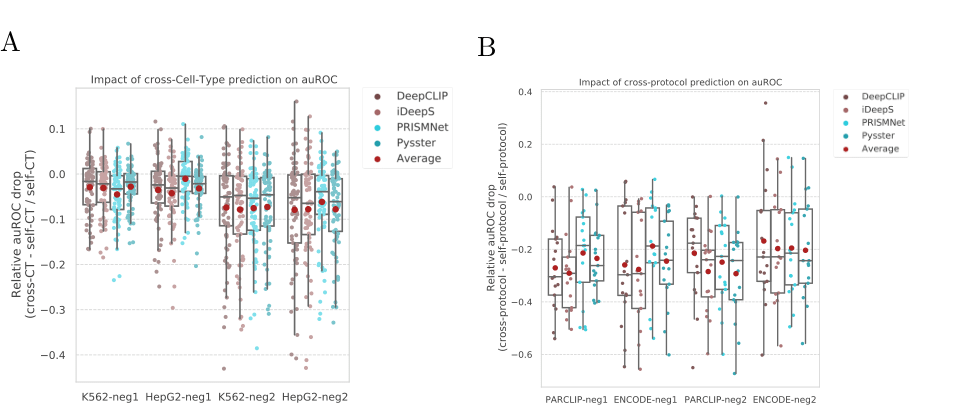


Supplementary Figure 3: Impact of cross-predictions as measured by relative auROC change from self- to cross prediction, for cross-cell-types predictions of 73 ENCODE RBPs (A) and 20 RBPs matched between ENCODE and PAR-CLIP (B). In (A) the average impact on performance in cross-cell type performance for K562-trained models and HepG2-trained models is respectively *−*0*.*0332 and *−*0*.*0298 in negative-1 setting and *−*0*.*0752 and *−*0*.*0741 in the negative-2 setting. In (B) the average impact on performance in cross-protocol performance for PARCLIP-trained models and ENCODE-trained models is respectively *−*0*.*2526 and *−*0*.*2421 in negative-1 setting and *−*0*.*2602 and *−*0*.*1911 in the negative-2 setting.
